# Electrochromic Device Demonstrator from Household Materials

**DOI:** 10.1021/acs.jchemed.2c00176

**Published:** 2022-09-22

**Authors:** Martin Rozman, Mojca Alif, Urban Bren, Miha Lukšič

**Affiliations:** † Centre for Functional and Surface Functionalized Glass, Alexander Dubček University of Trenčín, Študentská 2, SK-91150 Trenčín, Slovakia; ‡ Faculty of Chemistry and Chemical Technology, University of Maribor, Smetanova ulica 17, SI-2000 Maribor, Slovenia; § Faculty of Chemistry and Chemical Technology, University of Ljubljana, Večna pot 113, SI-1000 Ljubljana, Slovenia; ⊥ First High School in Celje, Kajuhova ulica 2, SI-3000 Celje, Slovenia

**Keywords:** General Public, High School/Introductory Chemistry, Demonstrations, Interdisciplinary, Hands-On Learning/Manipulatives, Electrochromism, Electrochromic Device, Electrochemistry, pH, Dyes/Pigments

## Abstract

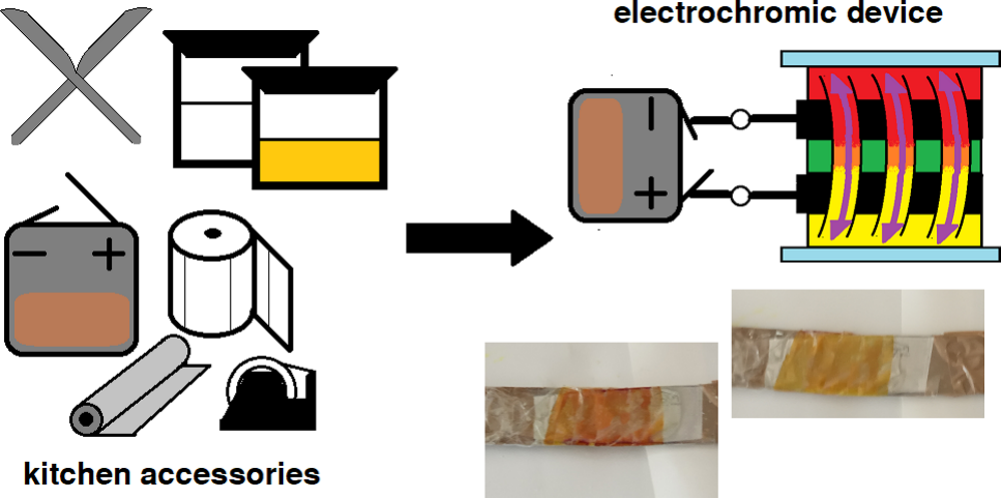

Electrochromism encompasses reversible changes of material’s optical properties (color, opacity) under the influence of an external electric current or applied voltage. The effect has been known for decades, but its importance continues to grow due to the rapid development of smart systems and the accompanying demand to build devices that consume less power. Most commercial electrochromic devices (ECDs) require sophisticated chemicals and advanced material preparation techniques. Also, the demonstration of electrochromism in chemistry classes mainly uses expensive WO_3_ films, intrinsically conductive polymers, and/or optically transparent electrodes (OTEs). The aim of this article is to present a simple and fast educational method to build ECDs from household materials without the need for OTEs: unsharpened kitchen knives are used as electrodes, curcumin from turmeric is used as the electrochromic dye, and baking soda is used as the electrolyte. The laboratory experiments presented will help students gain a deeper understanding of the fundamentals of electrochemistry (electrolysis, pH change) and electrochromism (in our case, color changes due to pH-induced keto-enol tautomerism of curcumin).

## Introduction

Electrochromism or electrochromic (EC) effect represents a behavior of a material which under an applied electric current or potential reversibly changes its color or opacity.^1,2^ A variety of metal oxides (e.g., WO_3_, MoO_3_, V_2_O_5_, TiO_2_, PbO_2_, Nb_2_O_5_) or hydroxides (e.g., Ir(OH)_3_, Ni(OH)_2_) form a class of inorganic EC materials, while viologens, quinones, phenazines, tertiary amines, pH indicators, and conjugated conducting polymers (e.g., polythiophenes, polypyrroles, polyanilines) belong to the class of organic EC materials. Various metal coordination complexes (e.g., with polypyridyls and phthalocyanines) that exhibit chromophoric properties exhibit EC effects, as well. Depending on the solubility of the redox states of the EC material, we can classify them into three types:^1−3^ surface-confined solid-state EC materials (solid in all redox states), electrodeposition EC materials (one redox state is soluble, while the other forms a solid film on the electrode surface), and solution-phase EC materials (soluble in all redox states). Once a redox state has been produced, the solid state and electrodeposition electrochromic devices (ECDs) retain it without additional charge supply (optical memory), while in the solution-phase ECDs, the electrochemically generated product diffuses away from the electrode and additional charge needs to be supplied until the whole solution is electrolyzed to achieve a complete coloration. Commercial examples of ECDs include information display elements, e-readers, smart windows, rearview intelligent mirrors, helmet visors, protective eyewear, indicator strips, camouflage and chameleonic fabrics, camera lens optical iris, etc.

Early ECDs were constructed using electrodes based on an optically transparent thin layer of metal oxides (e.g., SnO_2_ or ZnO_2_) doped to increase the electrical conductivity and durability of the material. Common examples of such optically transparent electrodes (OTEs) include fluorine-doped tin oxide (FTO),^4^ antimony-doped tin oxide (ATO),^5^ and tin-doped indium oxide (ITO)^6,7^ electrodes. Next generation OTEs followed the discovery of intrinsically conducting polymers (ICPs).^8,9^ The most promising ICPs exhibiting good electrical conductivity and optical transmittance properties come from a family of modified polythiophenes (e.g., poly(3,4-ethylenedioxythiophene) or PEDOT and poly(3,4-ethylenedioxythiophene):polystyrenesulfonate or PEDOT:PSS).^10−12^ At present, certain thin layer deposition methods such as sputter deposition,^13^ roll-to-roll techniques,^14^ and improvements in graphene production^15^ offer potential for lower ECD production costs, thus leading to new cheaper EC thin layers.^16,17^

To ensure the transport of ions between the two electrodes—required for balancing their charges—a layer of electrolyte (preferably of high ionic and low electronic conductivity) is used in all ECDs. The EC material is in solution-phase cells dissolved in either aqueous electrolyte solution or in a polar organic solvent (e.g., γ-butyrolactone, acetonitrile, dimethylformamide).

Even though ECDs can be relatively simple to assemble, several issues arise when using them as EC demonstration devices in scholarly applications. Didactic examples of ECDs employ expensive OTE materials, preparation of metal oxides at elevated temperatures, or use of hazardous metals such as lead or nickel and/or lithium salts.^18−24^ However, several types of simple solution-phase ECDs are known, which use pH indicators as an electrochromic dye.^2,25^ Their EC mixture consists of a pH indicator dye dissolved in, for example, aqueous solution of supporting electrolyte to increase its ionic conductivity, and the change in the pH near the electrodes due to water electrolysis causes the color change of the solution. These devices are suitable for educational purposes: they demonstrate the basic chemical principles (electrolysis, pH or redox indicators) as well as electrochromic aspects of materials (change in color) and develop practical skills of assembling simple functional ECDs. Note that in such decomposition-indicator ECDs, the current causes the color changes indirectly by pH changes of the solution containing the dye and not by a redox reaction of the dye itself.

Recently, we proposed a way to build an ECD without the need for OTE.^25^ We reuse its architecture here to provide a quick and simple way to assemble an ECD and test its function in chemical education. All materials are inexpensive and can be found in a majority of laboratories and households: unsharpened cutlery stainless steel kitchen knives as electrodes, curcumin (turmeric spice) as an EC material, aqueous solution of sodium bicarbonate (baking soda) as an electrolyte, kitchen paper towel as an EC mixture holder, office adhesive tape as an insulator and protector, kitchen aluminum foil as electric wires, office clips as holders, and alkaline disposal battery or cell phone battery as an external power source.

## Experimental Section

### Chemicals, Equipment, and Materials

All chemicals were obtained commercially from a food supply store or pharmacy: turmeric spice (Kotányi), baking soda (Kotányi), ethanol (Sigma-Aldrich, 96%), demineralized water, kitchen aluminum foil (Impol EN-AW-1200), white cellulose kitchen paper (Paloma Multi Fun XL 2/1), office adhesive tape (AERO PVC tape, 50MMX66M), insulating tape (Tesa, article no. 56192-10 and Scotch SCOTCH MAGIC TAPE 810, 3M), and office clips. Unsharpened stainless steel cutlery kitchen knives were used as metallic electrodes. A commercial 4.5 V battery (Varta longlife extra 3LR12 4.5 V BL/1) or a lithium-polymer battery from a Nokia C3-00 BL-5J cell phone was used as an external power supply. Titripur (Sigma-Aldrich) ready-to-use solutions of 0.1 mol dm^–3^ HCl and NaOH were applied. Details of the ECD component and solution preparations are provided in the Supporting Information (SI) (part I) and displayed in Figure S3. UV–vis measurements were conducted using a Cary 50 UV–vis spectrophotometer (Agilent, USA).

### ECD Assembly Procedure

Two identical clean unsharpened stainless steel kitchen knives served as electrodes. Each knife was first covered on one side of its metallic surface with insulating tape. The two knives were then placed on top of each other, so that the surfaces in contact were insulated by the tape. A piece of kitchen paper towel (impregnated beforehand with a turmeric spice solution in 50% aqueous ethanol and then dried) was wrapped around the two knives so that both metallic surfaces were covered. The paper was tightly pressed to the surface of the electrodes and secured with the office clips. The assembly was subsequently soaked with a 5% baking soda (NaHCO_3_) aqueous solution, so that the paper carrier was completely wet. The entire wet surface was then protected by transparent office adhesive tape, and the clips were removed. Two pieces (2 cm by 15 cm) of kitchen aluminum foil were rolled to create a tube (electric wire) and covered with insulating tape, except for the furthermost 5 mm on each side. One end of the first aluminum wire was attached with a paper clip to the metallic surface of one knife, and the other side of the wire was connected to the positive pole of the battery. In the same manner, the negative pole of the battery was connected to the other knife using the second aluminum wire. Both wires were kept away from each other in order to prevent the creation of a short circuit. (Alternatively, commercially available insulated electrical wires can be used, with alligator clips on both sides.) Details on the ECD assembly using knives are provided in the SI (I and II), and the ECD is displayed in Figure S4. A video demonstrating the construction of the ECD is available at https://video.arnes.si/en/watch/g8xlttgktt7s.

## Hazards

The chemicals used in the EC mixture (curcumin, water, baking soda) do not present serious hazards or safety risks. Ethanol is a flammable liquid, so contact to open fire should be avoided. During operation, the solution inside the ECD turns either acidic or alkaline and can cause skin irritation if a person comes in direct contact with it. In this unlikely case, it is advised to flush the skin with plenty of water for a minimum of 15 min. Curcumin can also cause color stains on hands or clothes, which can be again removed with water. Use of safety protective wear is mandatory (lab coat, safety goggles, and latex gloves). Due to gas formation during electrolysis, it is recommended to conduct the experiments in a well-ventilated area. Due to the risk of short circuits, which can cause fire, it is mandatory that the contacts and wires connecting the power supply and the ECD are properly insulated. Special caution must be taken if ECD is connected to lithium-ion or lithium-polymer battery power sources.

## Results and Discussion

The experiment was performed by a total of 16 students, two of whom were pursuing a second-cycle master’s program in chemical education and 14 high school (gymnasium) students between the ages of 16 and 18. The university students performed this experiment in 2019, and high school students in 2022. The basic topics underlying the proposed experiment are covered in the Slovenian national curriculum for chemistry in high schools (gymnasium). All students completed the experiment and obtained reliable results. They actively discussed each step with the teacher and were highly motivated while performing the laboratory exercise. At the end, they analyzed the results and solved the questionnaire (given in the SI (IV)).

A total of 4 class hours (4 times 45 min) was required to conduct the proposed laboratory experiment. The teacher/technician needed about 1 h to make all of the necessary preparations for the experiment. Before the students began the experimental work, the teacher explained to them the general idea of the experiment, i.e., the assembly of an electrochromic device in the *inverted* sandwich configuration, along with the necessary theory behind the experiment (acid–base theory, pH, indicator dyes, electrolysis, electrochromism). The teacher actively encouraged students to ask questions related to the topic and provided additional explanations to fill in gaps in their knowledge. Students were given notes with detailed instructions on how to conduct the experiment (given in the SI (II)) and were shown a short video demonstrating the lab work (https://video.arnes.si/en/watch/g8xlttgktt7s). After the 45 min theoretical introduction and discussion, students had about 15 min to prepare for the lab work. The main part of the experiment lasted 90 min (2 class hours). Students worked in groups of two or three. The teacher was present at all times during the lab work to help the students with any questions. After the students assembled the ECD, the teacher inspected the device. After the inspection, the students were allowed to begin testing the performance of the device. Students measured the active area of the device, the cycling times, and photographed the coloration states of the device. Students were instructed to perform at least two coloration changes. Upon completion of the experiments, students were instructed to disconnect the device from the power source (battery) and clean the laboratory equipment. The ECDs were disassembled later by the teacher. After all students had completed the experimental part, each of them was given a questionnaire to fill out on their own. The students had 20 min to complete the questionnaire. After that, a third phase of 10 min was allocated for checking the correct answers and for any questions the students might have about the subject. During the third phase, the teacher briefly reviewed the students’ answers and commented on the most common errors. This final post-experimental part lasted approximately 45 min.

Students did not receive a final grade for this laboratory experiment, but the teacher assessed their knowledge and performance in all phases: (i) In the initial theoretical phase, students were assessed based on their participation (answering and asking questions). (ii) In the main part of the experiment, students were assessed on how they followed the given instructions, how much they paid attention to details while assembling and testing the ECD, and how well they were able to predict the outcome and compensate for any mishaps during the experiment (e.g., loose transparent insulation tape or insufficient electrolyte, etc.). In addition, students were also evaluated at this stage on their ability to work as a team and complete various tasks during assembly and testing. (iii) In the post-experimental phase, students were assessed based on their answers in the questionnaire. The majority of students completed the questionnaire with one error or less (78.6%), whereas one of 14 students answered 3 of 8 questions incorrectly (SI (IV)). It should be noted that the high school students who participated in this laboratory experiment were highly motivated and had previously shown interest in natural sciences (chemistry, physics, and biology), having excellent grades in these subjects. It was also noted that the vast majority of students had no difficulty answering the questionnaire in English. During all phases, students were allowed to communicate freely with the teacher. Students were also encouraged to help each other and discuss the topic during the experimental phase. The students were satisfied with the knowledge they acquired on the demonstrated topic, putting the theoretical knowledge from school into practice. Since electrochromism is not part of the curriculum, students learned that certain materials can change color as a result of an external electrical stimulus and gained knowledge of real-life ECD applications. They were able to connect and integrate previously acquired knowledge about pH, indicators, water electrolysis, and acid–base equilibria into practice (hands-on device made with materials they may find in their household). They commented positively, praising the simplicity of the experiment and the possibility of quick troubleshooting.

**Figure 1:**
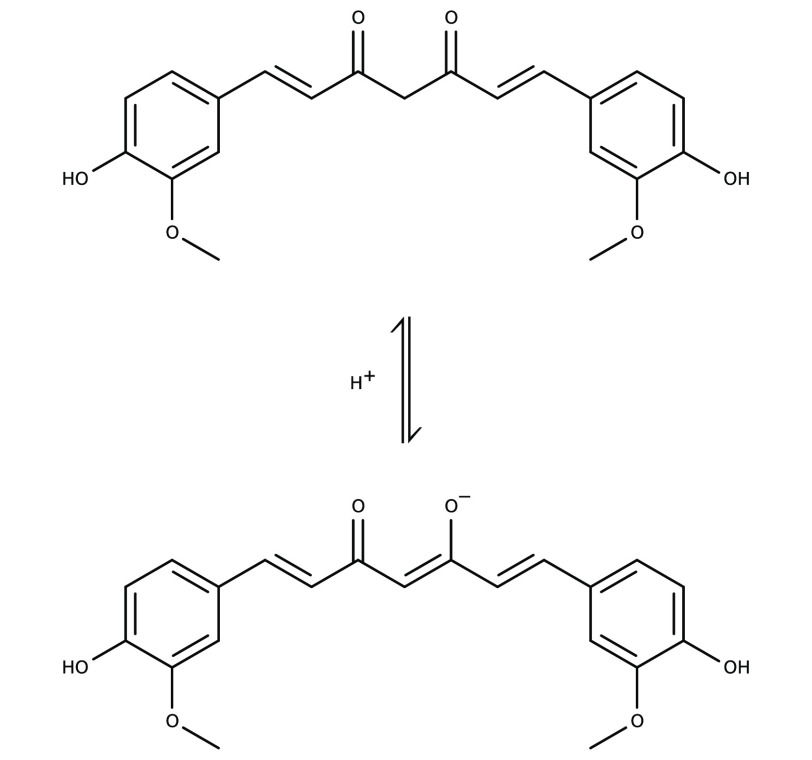
Tautomeric forms of curcumin: in acidic media, the predominant form is keto (top), while the enolate form (bottom) prevails in alkaline solutions. The color in acidic solutions is yellow to yellow-greenish and orange to orange-red in alkaline media (cf. Figure 2).

The presented ECD forms a decomposition-indicator cell. The change in the pH of the EC mixture (aqueous solution of sodium bicarbonate and turmeric spice) is through water electrolysis coupled with the chemical reaction of the pH indicator (curcumin from turmeric spice) producing the desired EC effect. Curcumin (IUPAC name (*E*,*E*)-1,7-bis(4-hydroxy-3-methoxyphenyl)-1,6-heptadiene-3,5-dione), a natural polyphenol, represents the yellowish pigment component of the turmeric spice. It exhibits keto-enol tautomerism (Figure 1): the molecule is in its keto form at neutral pH values and below (acidic media) and in its enolate form at higher pH values (alkaline media). Depending on the curcumin concentration, acidic solutions are colored yellow to yellow-green and alkaline solutions orange to orange-red.^26^ The color change of turmeric spice containing curcumin takes place in the pH range from 7.4 to 8.6.^27^

The electrochemical decomposition of water is well-documented:^28^ During the course of electrolysis, the pH gradually changes: water becomes acidic near the anode  and alkaline near the cathode (2H_2_O + 2*e*^–^ → H_2_ + 2OH^–^). In acidic media, the anodic reaction becomes , while the cathodic process is 2H^+^ + 2*e*^–^ → H_2_. In alkaline media, the anodic reaction becomes , while the cathodic process is . (In this paper, we do not differentiate between H^+^ and H_3_O^+^: H^+^ is very short-lived and forms H_3_O^+^ with water.) These pH changes of the curcumin solution near the two electrodes are, therefore, responsible for the keto-enol tautomerism and the concomitant coloration effect of the indicator dye.

**Figure 2:**
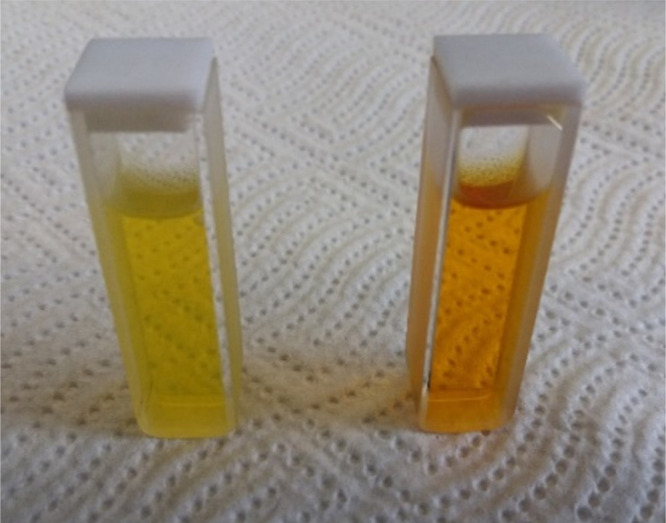
Solutions of turmeric spice in 0.1 mol dm^–3^ HCl (left) and in 0.1 mol dm^–3^ NaOH (right). Solutions were prepared by adding one drop of turmeric spice solution to 1 mL of 0.1 mol dm^–3^ acid or base.

In the first part of this demonstration, which is optional for high school students (the teacher can only demonstrate this part or show pictures/video), the student was introduced to the pH indicator properties of curcumin. He/she prepared the solutions of turmeric spice in aqueous 0.1 mol dm^–3^ hydrocloric acid (HCl) and in aqueous 0.1 mol dm^–3^ sodium hydroxide (NaOH). The pH values of HCl and NaOH solutions were also determined by the use of a universal indicator paper to 1 and 13, respectively. The student filled two 1 cm cuvettes, putting 1 mL of HCl solution into one and 1 mL of NaOH solution into the other as well as added one drop of turmeric spice solution into both of them (cf. SI (I and III)). Observing the color, the student concluded that in acidic media the solution was yellow and in alkaline media the solution was orange (see Figure 2). Using a UV–vis spectrophotometer, the student subsequently recorded the absorbance spectra of both solutions (Figure S1) and reflected on the relation between the absorption spectra and the color of the solution (cf. SI (I)).

**Figure 3:**
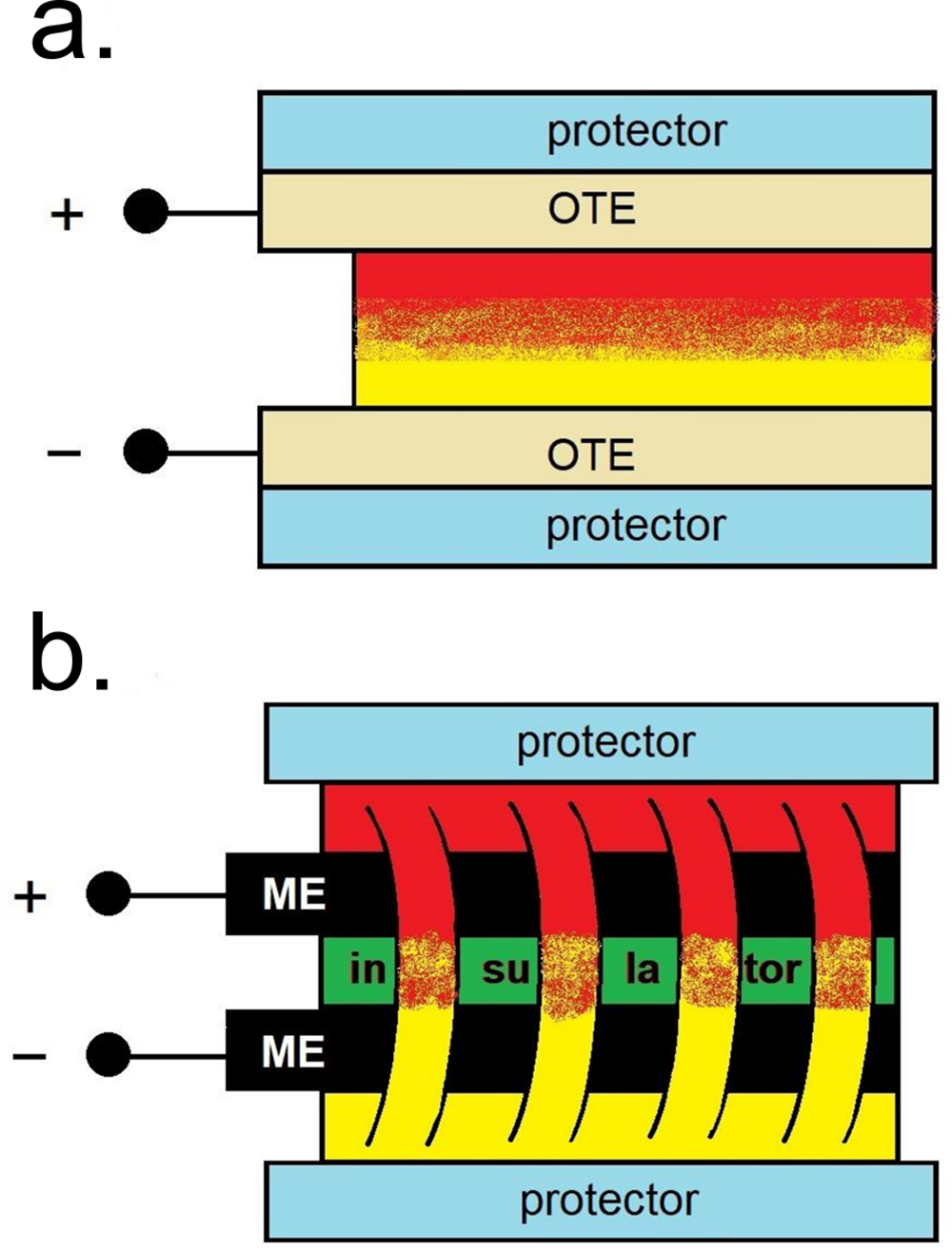
Two-dimensional schematics of an ECD in (a) a sandwich and (b) an *inverted* sandwich topology. In the sandwich topology, optically transparent electrodes (OTEs) are required, while in the inverted sandwich architecture, optically nontransparent metal electrodes (MEs) can be used. While in the sandwich ECD, the electrochromic (EC) mixture is sandwiched between the two OTEs, and the EC mixture is surrounding the electrodes in the inverted sandwich topology. Electrical insulation of the contact electrode surfaces is required to prevent short circuits, while the EC carrier facilitates the current flow. The cell is encapsulated within an optically transparent nonconducting protector. See also Figure S2 in SI (I).

The main part of this school demonstration formed the assembly of a simple ECD from household materials and testing its response. This device does not require the use of expensive optically transparent electrodes. The so-called *inverted sandwich* cell architecture, first introduced by us in ref (25), enables the construction of a reflectance electrochromic cell from two optically nontransparent metal electrodes. In contrast to the classical sandwich configuration (EC mixture is sandwiched between the two OTEs), its topology is inverted: the electrodes are sandwiched between the layer of the EC electrolyte carrier impregnated with the EC mixture (see Figure 3 and Figure S2). The role of the carrier is also to function as a continuous salt bridge on the edges of the ECD. The student was given instructions on how to use unsharpened stainless steel kitchen knives as electrodes. Attention was paid that he/she understands the cell’s topology and why the insulation of certain parts of the surface of the metal electrode is needed to prevent short circuits. The student used a kitchen paper towel as a carrier of the electrochromic mixture in this solution-phase ECD. He/she was then instructed on the flow of the electric current in this ECD and reflected on the importance of a salt bridge in electrochemical cells.

**Figure 4:**
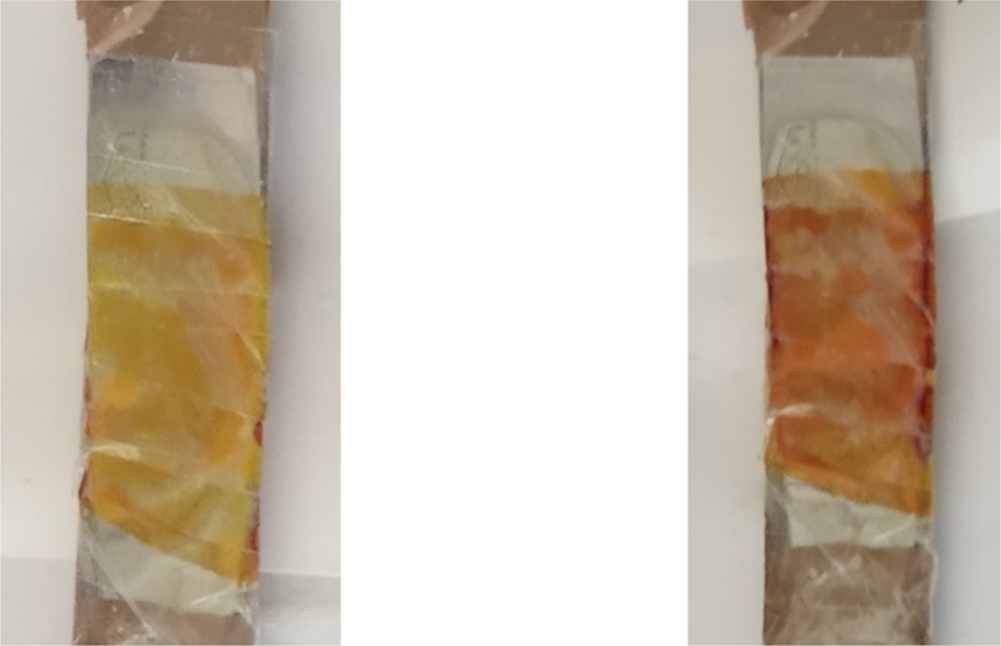
Photos of the ECD’s anode (left) and cathode (right) connected to a 4.5 V battery for 45 s. Unsharpened stainless steel kitchen knives were used as electrodes. States of the warm-up cycle are presented.

Applying wires made out of kitchen aluminum foil (or using commercially available insulated electrical wires equipped with alligator clips), the ECD was connected to a battery. Two different nominal voltages were used: a classical 4.5 V battery and a 3.7 V battery from a cell phone. Testing the performance of the ECD at various operating voltages allowed students to investigate the effect of the applied potential difference between the anode and cathode on the response time of the ECD (i.e., the time it takes for the color to change) and how the response time correlates with the working area of the electrode. A commercially available potentiostat with adjustable voltage can be used as an external power source instead of batteries. On the metal electrode (knife) connected to the positive pole of the battery (the anode), the solution gradually became acidic, whereas on the metal electrode connected to the negative pole of the battery (the cathode), the solution became alkaline (we call this the warm-up cycle of the ECD). The changes in the pH of the EC mixture were responsible for the color changes on the visible sides of the device: on the anode side, it gradually became more and more yellowish, whereas on the cathode side, it turned orange-brownish. In Figure 4, photographic images of both coloration states of a knife-based ECD are presented (see also Figure S4). The changes in color match those of pure solutions in Figure 2.

Using a stopwatch, the student also monitored the time required for the color of the visible sides of the ECD to change (the cycling time). He/she recorded the time of the warm-up cycle, i.e., the time needed for a color change when the cell was connected to the battery for the first time. When the coloration states were established on both electrodes, their polarity was reversed. The student concurrently measured the working time, i.e., the time to revert color from the acidic state to the alkaline state (or vice versa). Data for knife-based ECDs at 4.5 V are provided in Table 1 (see also Table S2 in SI (I)).

**Table 1:** Cycling Times (Warm-up and First Working Cycle) of ECDs Using Knives As Electrodes with a Given Working Surface Area

	knife-based ECD (4.5 V battery)
surface area (mm^2^)	360
warm-up cycle (s)	45
working cycle (s)	110

From these data, the student concluded that the working cycle is longer than the warm-up cycle. In the first working cycle, the pH initially changes back from the acidic (alkaline) state to neutral (from which the warm-up cycle started) and then increases (decreases) to the alkaline (acidic) state. To explore the effect of the electrode working area on the cycling times, the student also tested the performance of the knife-based ECD at 3.7 V (see Table S2 in SI (I)). From these data, the student concluded that for a given electrode working area, increasing the voltage speeds up the cycling times due to the accelerated water electrolysis. The students also recognized that increasing the voltage could lead to cell performance failure (uneven coloring, formation of burnt spots, etc.), while insufficient voltage could lead to color changes only at the edges of the device. These experiments highlight the need for parameter adjustment in optimization of the ECD performance.

The student was instructed to closely monitor if any bubbles formed during the ECD operation. This helped to discuss problems related to encapsulation of technological devices. It stimulated him/her to think of options to avoid gas formation, i.e., the use of alternative EC mechanisms which do not rely on pH changes due to the supporting electrolyte solution electrolysis.

## Conclusion

We have presented a simple electrochromic device assembled from inexpensive, readily available household materials. It demonstrates in an educational way that it is possible to construct ECDs that combine an advanced way of changing colors with simple tools and materials. The construction strategy is general enough that other chemicals and electrode materials can be used, e.g., alternative EC materials (viologens, azo dyes, and pH indicators (e.g., bromothymol blue)), alternative supporting electrolytes (e.g., sodium acetate), alternative materials with sufficiently high electrical conductivity for the electrodes, alternative suitable power sources, etc. It demonstrates to students the need for synergy between different fields of chemistry in solving technological problems that span the field of chemical engineering: in our case, knowledge of acid–base equilibria, pH indicators, general electrochemistry (electrolysis), and electrical properties of materials and corrosion. The constructed ECD can be used for teaching purposes and provides a visual representation of charge transfer and energy storage processes. By eliminating the need for expensive optically transparent electrodes, this architecture can be used in other applications such as solar cells, photodetectors, or even light-emitting diodes, but this comes with significant modification challenges compared to the current state of the art.^29^ Finally, the device architecture can be used as an electrochemical sensor in analytical chemistry because the surface near the selected electrode can be exposed to its environment.

## Supporting Information Available

(I) Required chemicals and materials alongside detailed experimental instructions to assemble and test the ECD; objectives taught and student’s reflections; (II) handouts for students to conduct the experiment; (III) separate student’s report on the ECD assembly and testing as well as (IV) student questionnaire; video showing the assembly and testing of the ECD is available at https://video.arnes.si/en/watch/g8xlttgktt7s (PDF)
