# Supplementary material for: Electrochromic Device Demonstrator from Household Materials
Source: J Chem Educ. 2022 Sep 22;99(10):3595–600. doi: 10.1021/acs.jchemed.2c00176 (PMC9558367; doi:10.1021/acs.jchemed.2c00176)
Supplement: Supplementary file 1 — ed2c00176_si_001.pdf [file ed2c00176_si_001.pdf]

Supporting Information (I) Electrochromic Device Demonstrator from Household Materials

Martin Rozman,[†,‡,¶] Mojca Alif,[§] Urban Bren,[‡] and Miha Lukšič[*,¶]

†*Centre for Functional and Surface Functionalized Glass, Alexander Dubček University of Trenčín, Študentská 2, SK-91150 Trenčín, Slovakia*

‡*Faculty of Chemistry and Chemical Technology, University of Maribor, Smetanova ulica 17, SI-2000 Maribor, Slovenia*

¶*Faculty of Chemistry and Chemical Technology, University of Ljubljana, Večna pot 113, SI-1000 Ljubljana, Slovenia*

§*First High School in Celje, Kajuhova ulica 2, SI-3000 Celje, Slovenia*

E-mail: miha.luksic@fkkt.uni-lj.si

Phone: +386 (0)1 479 8539. Fax: +386 (01) 2419 144

Objective

The objective of this handout is to comprehend the following processes of assembling and testing a simple electrochromic device (ECD) demonstrator: color changes of the indicator dye induced by pH variation, water electrolysis as a means to influence the solution's pH value, basic concepts of the electrochromic effect, the basic architecture of the inverted sandwich ECD and quantifying the performance of the ECD in terms of cycling (response) times. Optionally, UV-VIS absorption spectroscopy can be used to quantify the color changes of the solution.

Experimental Overview

A. Assessing the Color States of the Indicator Dye

Under the guidance of the teacher, the students prepare the solutions of turmeric spice powder in acidic and alkaline media and monitor their colors. Optionally, they record their UV-VIS absorption spectra and correlate their color to the absorption maxima of both states.

The required chemicals and equipment:

0.1 $\mathrm{mol\,dm^{-3}}$ aqueous HCl solution
0.1 $\mathrm{mol\,dm^{-3}}$ aqueous NaOH solution
ethanol (96%)
turmeric spice (curcumin)
four 100 mL beakers
two 1 cm & 1.5 mL cuvettes (or centrifuge tubes)
a spoon, spatula, or glass rod
a dropper
a laboratory balance (or measuring spoons set)
pH test strips (0-14)
a UV-VIS spectrophotometer [for optional experiment]

The student first determines the pH values of a 0.1 $\mathrm{mol\,dm^{-3}}$ aqueous HCl and NaOH solutions using a universal indicator pH test strip. He/she finds out that the HCl solution has pH $\approx$ 1, and NaOH solution has a pH $\approx$ 13.

Next, the student prepares the water-ethanol solution of curcumin (turmeric spice). To 25 mL of ethanol and 25 mL of demineralized water 0.5 g of turmeric spice are added (a 100 mL beaker can be used). The solution is stirred for one minute and left to rest for 5 minutes. This allows for any undissolved particles to settle on the bottom of the beaker. The solution is subsequently decanted into a clear 100 mL beaker.

Approximately 1 mL of 0.1 $\mathrm{mol\,dm^{-3}}$ aqueous HCl is put into the cuvette (or plastic centrifuge tube). Using a dropper, one drop of decanted turmeric spice water-ethanol solution is added and mixed. An analogous procedure takes place in the second cuvette (or plastic centrifuge tube) with the 0.1 $\mathrm{mol\,dm^{-3}}$ NaOH solution.

The student observes a contrast in the color of acidic and alkaline solutions of turmeric spice. Different coloration states are discussed in the light of the keto-enolate tautomerism of the curcumin, caused by differences in the pH values of the two solutions (see Figure 1 of the main paper).

Optionally: To quantify the effect, the absorbance spectra of the acidic and alkaline turmeric spice solutions are recorded in the range of wavelengths from 300 to 800 nm. A Cary 50 UV-VIS spectrophotometer was applied. A 1.5 mL cuvette with 1 cm optical path was used and the baseline correction was performed with a 0.1 $\mathrm{mol\,dm^{-3}}$ HCl (or NaOH).

From the recorded spectra shown in Figure S1, the student determines the wavelengths of the absorption maxima of the acidic and alkaline turmeric spice solutions. He/she then correlates the wavelength of the absorbed light with the color of the solution through the concept of complementary color.

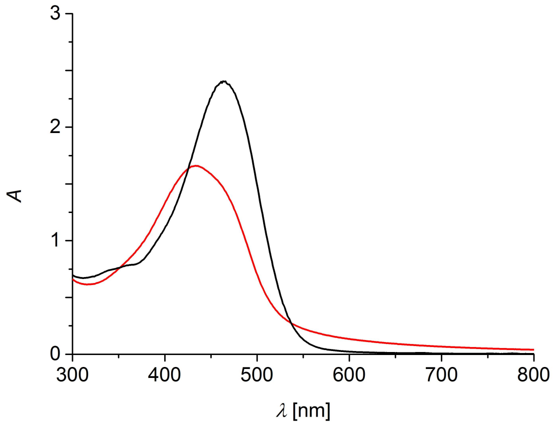

Figure S1: UV-Vis spectra (absorbance, A, vs. wavelength, λ) of solutions of turmeric spice in 0.1 $mol\,dm^{-3}$ HCl (red curve) and in 0.1 $mol\,dm^{-3}$ NaOH (black curve). Spectra were recorded at room temperature.

The student can use an on-line *Wavelength to Colour* converter to extract the Hex color code corresponding to the two absorption maxima wavelengths:

https://academo.org/demos/wavelength-to-colour-relationship/

Next, he/she applies the on-line *Color Calculator* to find the corresponding complementary colors:

https://www.sessions.edu/color-calculator/

In our case, the absorption maxima, the corresponding Hex color codes and the codes of the complementary colors are provided in Table S1 (see also Figure 2 of the main paper).

Table S1: The wavelength of the absorption maximum, λ_{max}, in the corresponding Hex color code and the complementary color's Hex code for the acidic and alkaline turmeric spice solution. The color of table cells corresponds to the given color code.

	λ_{max} [nm]	Absorbed color Hex code	Complementary color Hex code
acidic solution	434	#2800FF	#FF9500
alkaline solution	463	#0089FF	#FF5E00

⇑ solution's color

B. Assembling the ECD

Under the guidance of the teacher, the students prepare the components of the ECD (electrodes, electrochromic mixture) and assemble the device in the so-called *inverted sandwich* topology.[1] They test the ECD performance by measuring cycling (response) times.

1. Basic Components of the ECD

The presented ECD demonstrator consist of two optically non-transparent metal electrodes, electrical insulation between the contact surfaces of the electrodes, holder of the electrochromic (EC) mixture – composed of an electrochromic dye in an aqueous electrolyte solution – covering both electrodes, and a transparent protector which covers the outer part of the device. Schematics of the *inverted sandwich* ECD assembly is provided in Figure S2.

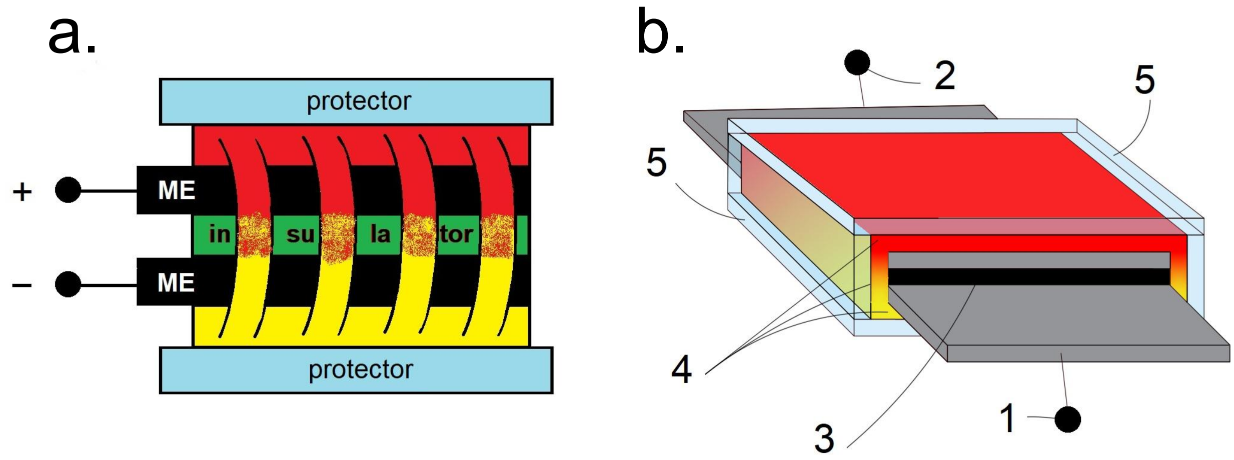

Figure S2: 2D (a) and 3D (b) schematics of the main components of the ECD in the *inverted sandwich* topology. Two metal electrodes (ME; 1 & 2) are on the contact surface insulated by a layer of an electrical insulator (3). The outer metallic surface is wrapped in an electrochromic mixture carrier (4), which also functions as a salt bridge enabling the flow of the electric current. The device is protected by an optically transparent non-conducting protector (5). See also Figure 3 of the main paper.

At this point, students discuss the composition of the device with the teacher. They understand why optically transparent electrodes (OTEs) are not required in the *inverted sandwich* configuration. They discriminate between transmission and reflectance devices. They assign the electric current flow direction in the device. They reflect on the role of the EC carrier to function also as a salt bridge. They understand why the solution at each side of the ECD changes its pH value and that this yields coloration differences at both visible sides of the ECD. They discuss which chemical processes are taking place at the anode and cathode.

In this demonstration two identical non-sharpened stainless steel kitchen knives are used as metal electrodes. The EC mixture holder is a general-purpose white kitchen paper towel. Insulation tape is applied to electrically insulate the contact surfaces of the two electrodes and the connecting wires, while the ordinary transparent office adhesive tape serves as a protector (device encapsulation). The connecting wires are made out of kitchen aluminum foil. Battery represents the external power source. Office paper clips are used to secure the connections between the battery and the assembled device. The EC mixture is turmeric spice dissolved in aqueous baking soda ($NaHCO_3$) solution. The electorhromic material is curcumin where keto and enolate forms yield different coloration states, and the equilibrium between both forms is regulated by the solution's pH value.

The required chemicals and materials for ECD assembly are listed below and depicted in Figure S3:

Electrodes	two non-sharpened stainless steel kitchen knives
Electrical insulator	insulating tape
Protector	transparent office adhesive tape
Electrolyte carrier	white kitchen paper towel
Wires	kitchen aluminum foil, or
	commercial insulated electrical wires with alligator clips
Connectors	office paper clips
Power source	commercial 4.5 V battery, or
	Li-polymer battery from the Nokia C3-00 BL-5J cell-phone, or
	commercial potentiostat
Electrolyte	baking soda ($NaHCO_3$)
Solvents	demineralized or distilled water
	ethanol (96%)
Electrochromic dye	turmeric spice, curcumin

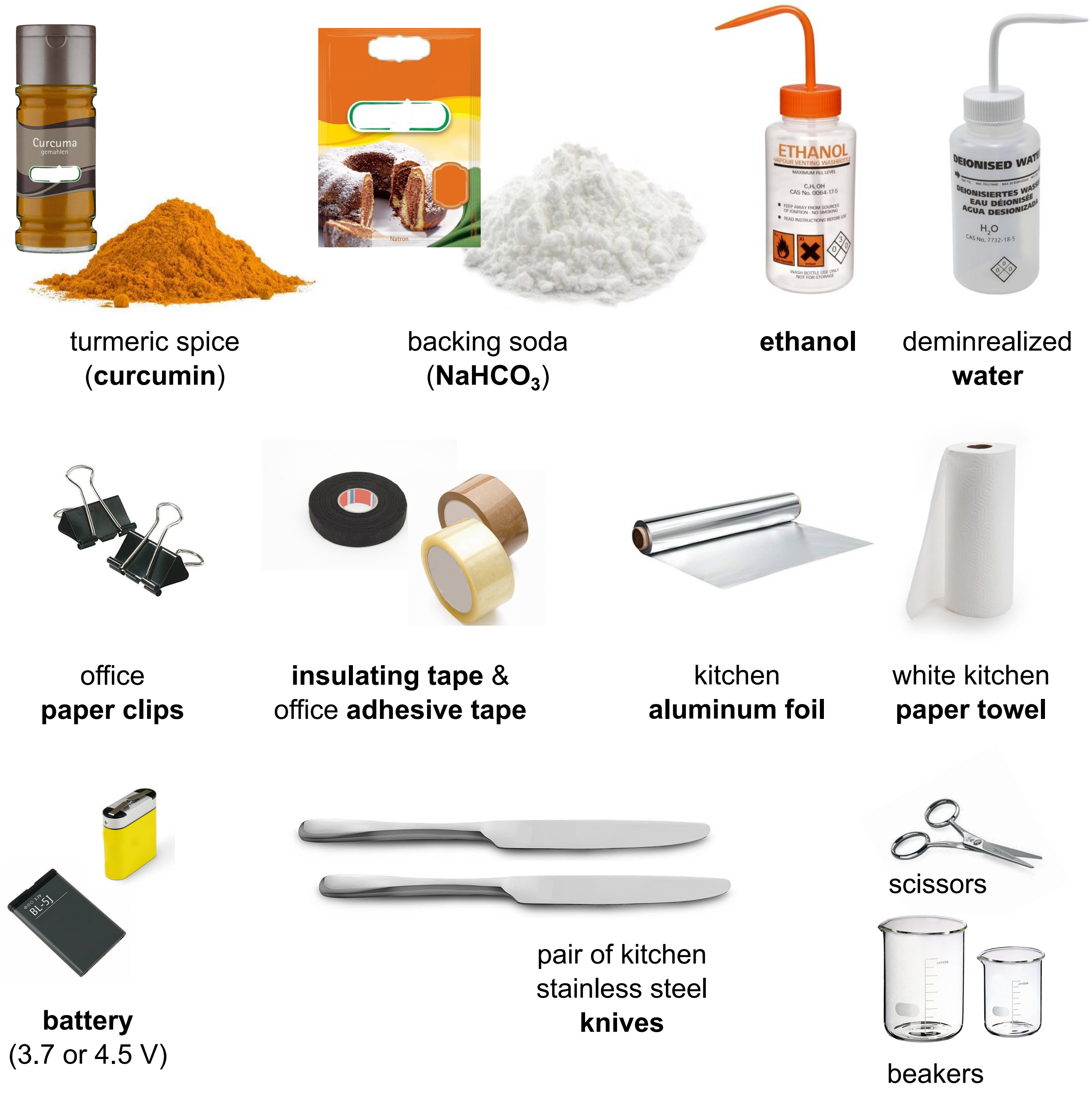

Figure S3: The basic components needed for an ECD assembly based on knife electrodes and curcumin as an electrochromic dye.

2. Preparation of the Supporting Electrolyte Solution, the EC Dye Solution, and the EC Mixture Carrier

In this step the student prepares all the required solutions and impregnates the EC carrier with an electrochromic mixture composed of curcumin from the turmeric spice and aqueous sodium bicarbonate (baking soda, $NaHCO_3$). Ions from $NaHCO_3$ function as an electrolyte, and curcumin as an electrochromic dye (pH indicator). The EC carrier (white kitchen paper towel) also serves as a continuous salt bridge.

The required chemicals and equipment are listed below:

two 100 mL beakers, one 50 mL beaker
Petri dish (15 cm or larger in diameter)
spoon, spatula, or glass rod
white kitchen paper towel
baking soda
turmeric spice (curcumin)
demineralized water (100 mL)
ethanol (96%)
laboratory balance (or measuring spoons set)
scissors
ruler
hair dryer

Preparation of the supporting electrolyte solution
The electrolyte solution is prepared in a 100 mL beaker by dissolving 5 g of baking soda in 100 mL of demineralized water. Water for ironing or car batteries can be used. The solution is then mixed with a spoon for 1 minute, or for as long as the CO_2 bubbles are present in the solution. Since certain baking soda powders include added excipients, any undissolved residue can be removed by filtering the solution through a piece of paper towel.

Preparation of the electrochromic dye solution
The electrochromic dye solution is prepared in a 50 mL beaker by mixing 0.5 g of turmeric spice with 25 mL of ethanol and 25 mL of demineralized water. Acetone (nail polish) can be used instead of ethanol. The solution is then mixed with a spoon for one minute, and left to rest for 5–15 additional minutes. This allows for any undissolved particles to settle on the bottom of the beaker. The solution is then decanted into a clear 100 mL beaker. (If part A. is performed, the same solution can be used in the ECD assembly.)

Preparation of the EC mixture carrier (paper impregnation)
A piece of white kitchen paper towel is cut in dimensions of 4 cm by 15 cm. It is completely soaked in the Petri dish or in the beaker with the EC dye solution. The paper is then carefully taken out of the solution, spread on a flat surface, and dried with a hair dryer. When dry, the paper with the adsorbed EC dye is cut into two pieces with dimensions of 2 cm by 15 cm.

3. Device Assembly

In this step the students are ready to assemble a simple ECD. All the required chemicals and materials are listed in subsections 1. and 2.

ECD with non-sharpened stainless steel kitchen knives as electrodes
Two identical clean non-sharpened stainless steel kitchen knives are used. One side of the cutting surface of both knives is first electrically insulated by covering the whole flat metal surface (up to the knife holder) with a layer of an adhesive insulation tape. The excess insulation tape is cut away with scissors. The insulated surfaces of the two knives are placed on top of each another (while the knife holders facing opposite sides). On each side, the insulation tape is used to secure the position of the assembly (tip of one knife is connected to the holder of the other knife and vice versa).

The piece of an EC mixture carrier (2 cm by 15 cm) is then rolled across the exposed (non-insulated) metal surfaces of the assembly, i.e. between the insulated ends of the assembly (Figure S4). It is important that the paper is pressed firmly onto the metal surface of the knives. This will form the visible area of the electrochromic device (the part where the color changes will take place). The contact between the paper carrier and the metal surface is secured with an office paper clip. The knive assembly with the clip attached is then carefully rinsed with supporting electrolyte solution above a sink or using a dropper. After the EC carrier has been completely soaked, the transparent adhesive office tape is tightly applied and slowly rolled over the assembly, starting at one of the insulated ends and proceeding to the other. While applying the transparent office tape (protector) the office paper clip is removed. The electrochromic device is now prepared for connection with the power supply (battery).

4. Connecting the ECD to an External Power Supply

In this step, the student makes its own electric wires that will help him/her to connect the assembled ECD with an external power source (battery). As an alternative, commercially available insulated electrical wires can be used, with alligator clips on both sides.

Electric wires are made from kitchen aluminum foil. Two pieces of the foil are cut with scissors in dimensions of 2 cm by 15 cm. Each of them is rolled as to create a cylinder, functioning as a wire. Insulation tape is used to cover the entire surface of the wire, except for furthermost 0.5 cm at each end. One wire is pressed to the positive channel of the battery, while the remaining exposed aluminum at the channel is secured with a paper clip. The second wire is pressed to the negative channel and secured in an analogous way as the first one. Both connecting wires are kept apart as to prevent short circuits.

We used two batteries: the first was a commercial 4.5 V battery (Varta longlife extra 3LR12 4.5 V BL/1) and the second was a lithium-polymer battery from Nokia C3-00 BL-5J cell-phone. It is only important that the power source is above the overvoltage of the electrolysis process. Other sources of DC electrical power can also be used. The optimal working voltage also depends on the active working surface area of the electrodes. If larger or smaller working surfaces are used, as in this experiment, the teacher should determine the optimum voltage before the students begin the experiment.

Next, the wires are pressed directly to the assembled ECD and secured with office paper clips. The wire connected to the positive pole of the battery is touching the holder of the first knife, and the wire connected to the negative pole of the battery is touching the second knife. Photographic image of the ECD connected to the power source is presented in Figure S4.

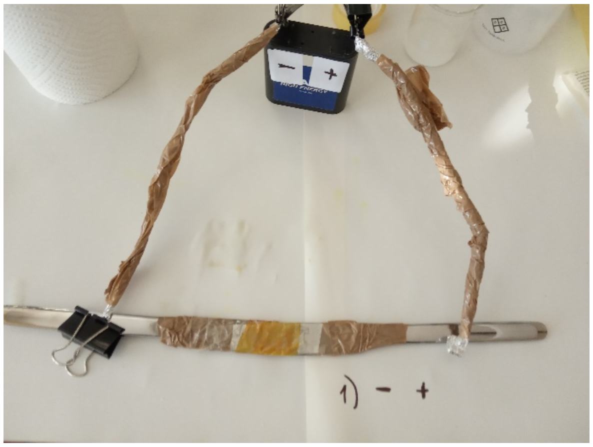

Figure S4: Picture of the assembled ECD with non-sharpened knives as electrodes, connected to an external power source (battery) via aluminum foil wires. A 4.5 V battery is used as the power source.

C. Assessing the Performance of the Assembled ECD

Under the guidance of the teacher, the students asses the performance of the assembled ECD by monitoring the color changes of the device and measuring cycling (response) times. Optimal working condition was 4.5 V for a knife-based ECD.

The operation of the ECD was checked by visually inspecting how the color of the observable surface of the electrodes changes with time and with the polarity of the electrodes (anode, cathode). The time needed to achieve a desired coloration state was measured with a stopwatch. The working surface area of the ECD was estimated by measuring its length and width by a ruler. The students were told to tabulate the measurements.

The surface of the knife connected to the positive pole (anode) remains yellow with time, while the surface of the knife connected to the negative pole (cathode) of the battery gradually changes its color to orange-brownish (see also Figure 4 of the main paper). The time required to change the color for the first time (the so-called warm-up cycle) is 45 seconds for knife-based ECD at 4.5 V. To change the color of the surface again, the wire connections are switched (the polarity of the electrodes is reversed). The time required to change the color the second, third etc. time (the so-called working cycles) is 110 seconds for the knife-based ECD.

The students are advised to observe any gas bubbles formation on the visible surfaces during the operation of the ECD. If the device experiences difficulties to switch color, pressure

with the office paper clip or pencil may be applied to the visible area, while the device is connected to the power supply. In general, the wider the metal electrodes, the longer the time required to sufficiently change the coloration of the device.

A demonstration of the cell perfomnace was made also at 3.7 V. The students observe that in the case of larger voltage of the battery (4.5 V) the cycling times are shorter compared to the battery with lower voltage (3.7 V). The students also realize that for a knife-based ECD the voltage of 3.7 V is not optimal: eventhough the warm-up and working cycle times were more than 3-times longer compared to the ECD operation at 4.5 V (Table S2), the working area got colored mainly on the cell edges. This demonstration helped students realize the importance of the optimization procedure for the best performance of a given ECD.

Students were also given the predetermined pH values of the EC mixture carrier of the ECDs at the end of the cycle: the anode side pH $\approx$ 6.5, the cathode side pH $\approx$ 12. They compared the colors with the colors of bulk solutions of turmeric spice in HCl and NaOH (*cf.* Figure 2 in the main paper).

Table S2: Cycling times (the warm-up and first working cycle) of the ECDs using kitchen knives as electrodes. ECDs with a given working surface area were connected to a 3.7 V and 4.5 V battery. Yellow color indicates conditions of ECD's optimal performance.

ECD<br>Power source	Knife-based<br>3.7 V battery	Knife-based<br>4.5 V battery
surface area [mm^2]	360	360
warm-up cycle [s]	150	45
working cycle [s]	330	110

At the end, the students discuss with the teacher the operational properties of the device. They correlate performance (cycling times) with the size of the device and the external voltage. They record the problems encountered during the assembly and operation† (mainly stemming from encapsulation and gas bubble formation). They discuss troubleshooting. They reflect on options to use alternative pH indicators or electrodes in the inverted sandwich ECD topology.

†The conditions under which the proposed cell is operating do not pose a serious risk for electrode corrosion. Information on which oxides of the metal elements forming the electrode are stable under certain pH conditions is given by Pourbaix diagrams.[2] These diagrams can be used to study the corrosion mechanisms as a function of pH, temperature, and concentrations of the reacting species. However, changing any of these parameters will result in a different diagram. Moreover, in the case of alloys (in our case, stainless steel), there are significant limitations in estimating the corrosion potential of metals.[3] It should be noted that the composition and microstructure of different austenitic stainless steels (e.g., the general-purpose chromium-manganese-nickel stainless steel type 202 or the commonly used chromium-nickel stainless steel types 304[4] or 316) lead to different Pourbaix diagrams.

Troubleshooting the Electrochromic Device

Constructed ECDs can under-perform under certain conditions, some of which are collected here:

- If the office adhesive tape is loosely glued around the device, the electrolyte holder might not have the required contact with the electrodes and the visible surface of the device will exhibit non-uniform coloration. The solution is to apply pressure on the top of the visible side with an office paper clip, pencil or thick glass rod.
- If the time between construction and testing of the device is too long, the device might get dry and can thus under-perform or will not even change color at all. The solution is to wet the EC carrier with the electrolyte solution.
- If the device has not started to change color after few seconds this could imply that the power supply is exhausted or there is a short circuit. Remove the device from the power supply and gently press on the visible area. If it feels that the area has ambient temperature, this indicates that power supply might be empty, however if the pressed area is hot, this indicates the possible presence of a short circuit. In the case of short circuits, the solution is to reassemble the device.
- If no color changes are observed for an extended period of time (a few minutes), the voltage of the external power source may be too low for the device. The voltage must be increased to create optimal working conditions. On the other hand, if the cell is burned out and stops working after the first cycle, the operating voltage is too high and must be reduced.
- If no color changes are observed, the cause may be the electrochromic solution of the turmeric dye. Some turmeric spices contain buffer species that prevent changes in the pH of the solution. It is recommended to use dried turmeric spices for cooking.

References

(1) Rozman, M.; Gaberšček, M.; Marolt, G.; Bren, U.; Lukšič, M. An Inverted Sandwich Electrochromic Device Architecture Does Not Require Optically Transparent Electrodes. *Adv. Mater. Technol.* **2019**, *4*, 1900389.

(2) Jones, D. *Principles and Prevention of Corrosion.*; Macmillan Publishing Company: New York, 1992.

(3) Cubicciotti, D. Potential-pH Diagrams for Alloy-Water Systems Under LWR Conditions. *J. Nucl. Mater.* **1993**, *201*, 76–183.

(4) Wang, S.; Sun, M.; Xu, Y.; Long, K.; Zhang, Z. Enhanced Localized and Uniform Corrosion Resistances of Bulk Nanocrystalline 304 Stainless Steel in High-Concentration Hydrochloric Acid Solutions at Room Temperature. *J. Mater. Sci. Technol.* **2018**, *34*, 2498–2506.

Supporting Information (II)
Electrochromic Device Demonstrator from Household Materials

Instructions for students to assemble and test knife-based ECD

Example video using knives:
https://video.arnes.si/en/watch/g8xlttgktt7s

SOLUTION PREPARATION

Supporting electrolyte solution

Weight 5 g of baking soda. Add 100 mL of demineralized water to a 100 mL beaker, then add 5 g of baking soda. Mix with a spoon or glass rod until all solute is dissolved.

Electrochromic dye solution

Weight 0.5 g of turmeric spice. Add 25 mL of water and 25 mL of ethanol to a 50 mL beaker, then add 0.5 g of turmeric spice. Mix the solution with a spoon or glass rod for one minute and let the solution rest for 5-15 minutes. Decant the clear solution into a clean 100 mL beaker.

EC mixture carrier (paper impregnation)

Cut a piece of white kitchen paper towel in dimensions of 4 cm by 15 cm. Add electrochromic dye solution into a Petri dish and soak the paper. Carefully take the paper out of the solution, spread it on a flat surface, and dry it with a hair dryer. Cut the dry paper into two pieces with dimensions of 2 cm by 15 cm.

DETERMINING THE COLOR OF THE CURCUMIN IN ACID AND ALKALINE MEDIA

Put about 1 ml of 0.1 mol/L HCl in a plastic tube and 1 ml of 0.1 mol/L NaOH in another plastic tube. Using a universal pH test strip, determine the pH of the 0.1 mol/L acid and base. Record the result in Table S3.

Next, add 1-2 drops of the electrochromic dye solution to each of the solutions and record the color in the Table S3.

Table S3: pH of 0.1 mol/L HCl and NaOH and the color of solutions with added dye.

	pH	Color of solution with added dye
0.1 mol/L HCl		
0.1 mol/L NaOH		

DEVICE ASSEMBLY

Electric wires (Optional)

Cut two pieces of kitchen aluminum foil in dimensions of 2 cm by 15 cm. Roll each piece to create a cylinder. Cover the entire surface of the wire (except for furthermost 0.5 cm at each end) with insulation tape.

Instead of home made wires, commercial insulated wires with alligator clips can be used.

Electrodes

Insulate one side of the cutting surface of two knives by covering the whole flat metal surface (up to the knife holder) with a layer of an adhesive insulation tape. Cut the excess insulation tape away with scissors. Place the insulated surfaces of the two knives on top of each another (knife holders facing opposite sides). Secure the position of the assembly on each side with insulation tape (tip of one knife is connected to the holder of the other knife and vice versa).

Device

Roll a piece of an EC mixture carrier (2 cm by 15 cm) across the exposed (non-insulated) metal surfaces of the assembly (between the insulated ends of the assembly). It is important that the paper is pressed firmly onto the metal surface of the knives. Secure the contact between the paper carrier and the metal surface with an office paper clip. Use a dropper to soak the paper EC mixture carrier with supporting electrolyte solution (baking soda solution). After the EC carrier has been completely soaked apply transparent adhesive office tape (start at one of the insulated ends and proceed to the other). Remove the office paper clip while applying the transparent office tape (protector).

ASSESSING THE PERFORMANCE

Measuring the active surface area of the device

Using a ruler, measure the horizontal (x) and vertical (y) dimensions of the active observable area of the device. Calculate the surface area ($A = x \cdot y$). Add the number into Table S4.

Connecting to power supply

Attach one aluminum wire (or commercial wire) to one knife holder of the device and the other wire to the second holder of the device. Secure with office paper clips. The other ends of the wire will be connected to the battery.

Device operation, cycling times

Write the color of the observable surface of the device into Table S4.

Attach one wire to the positive pole of the 4.5 V battery, and the other wire to the negative pole of the 4.5 V battery (other voltages can be tested too). Secure with office paper clips. When the second wire is attached, start measuring time with a stop-watch. Observe the color changes. Report time needed to change the color of the observable area into Table S5 (warm-up cycle). Report also the initial and final color.
Reset the stopwatch. Switch the polarity of the electrodes. Again, measure time for the observable surface to change color. Report the initial and final color and working cycle time into Table S5.

Table S4: Dimensions of the active surface and initial color of the observable surface

Surface area [mm^2]	
Initial color	

Table S5: Cycling times of ECD and colors of observable surface (external power voltage: ________ V)

	Time [s]	Initial color	Final color
Warm-up cycle			
Working cycle			

Describe any additional phenomena during color change (formation of bubbles, uneven color at the borders…)

Comments:

Supporting Information (III)
Student's Report on the
Electrochromic Device Demonstrator
from Household Materials

April 11th, 2022

Electrochromism represents an effect where a material or a device starts to change color when exposed to electric current or voltage. Unlike liquid crystal displays, this newly obtained color is stable even after electric charge has been terminated. Electrochromic devices have multiple applications, such as dimmable glass and mirrors and can also be used as electronic ink displays in e-readers. In this report we tested how an electrochromic device can be easily constructed from household components.

1 Objective

The objective of this laboratory experiment was to construct a simple electrochromic device and test its performance. The basic parts of the electrochemical cell were assembled from the materials that can be readily found in a majority of households or can be purchased in general stores and pharmacies.

2 Materials & Chemicals

Materials:

- two non-sharpened stainless steel kitchen knives
- a roll of white kitchen paper towels
- a roll of kitchen aluminum foil
- an insulating tape
- a transparent office adhesive tape
- office paper clips
- scissors
- a spoon

- a dropper
- a ruler
- a kitchen balance
- a hair dryer
- a stopwatch
- two 100 mL and one 50 mL beaker
- one 25 mL graduated cylinder
- two plastic centrifuge tubes (1.5 mL)
- pH strips (range 0-14)
- a 4.5 V alkaline battery

Chemicals:

- baking soda
- turmeric spice
- demineralized (ironing) water
- ethanol (96%)
- aqueous 0.1 mol/L hydrochloric acid
- aqueous 0.1 mol/L sodium hydroxide

3 Solution Preparation

Electrolyte solution: 100 mL of demineralized water was put into a 100 mL beaker and 5 g of baking soda were added. The solution was stirred until all the solute completely dissolved.

Turmeric spice solution: 25 mL of ethanol and 25 mL of demineralized water were put into 50 mL beaker and 0.5 g of turmeric spice was added. The solution was stirred for approximately 1 minute and then left to rest for another 5 minutes. It was carefully decanted into a clean 100 mL beaker.

EC mixture carrier preparation: A 4 cm $\times$ 15 cm piece of paper towel was cut and carefully placed into the turmeric spice solution, where it was left to soak for 1 minute. The paper was taken out of the solution, spread onto a clean working surface and slowly dried with a hair drier. When dry, it was cut into two stripes of 2 cm $\times$ 15 cm.

4 Determining the Color of the Turmeric Spice Solution in Acidic and Alkaline Media

The pH values of the 0.1 mol/L HCl and NaOH aqueous solutions were determined by universal indicator pH strips. One strip was placed into the acidic and the other into the alkaline solution for a few seconds. The color of the strip was then compared with the enclosed color chart (Figure S5).
pH values of the HCl and NaOH solutions were indeed determined at 1 and 13, respectively.

Approximately 1 mL of 0.1 mol/L HCl solution was put into a plastic tube and one drop of turmeric spice solution was added with a dropper. In yet another plastic tube 1 mL of 0.1 mol/L NaOH solution was again mixed with one drop of turmeric spice solution. The color of the acidic solution was yellow, and the color of the alkaline solution was orange-red (Figure S5).

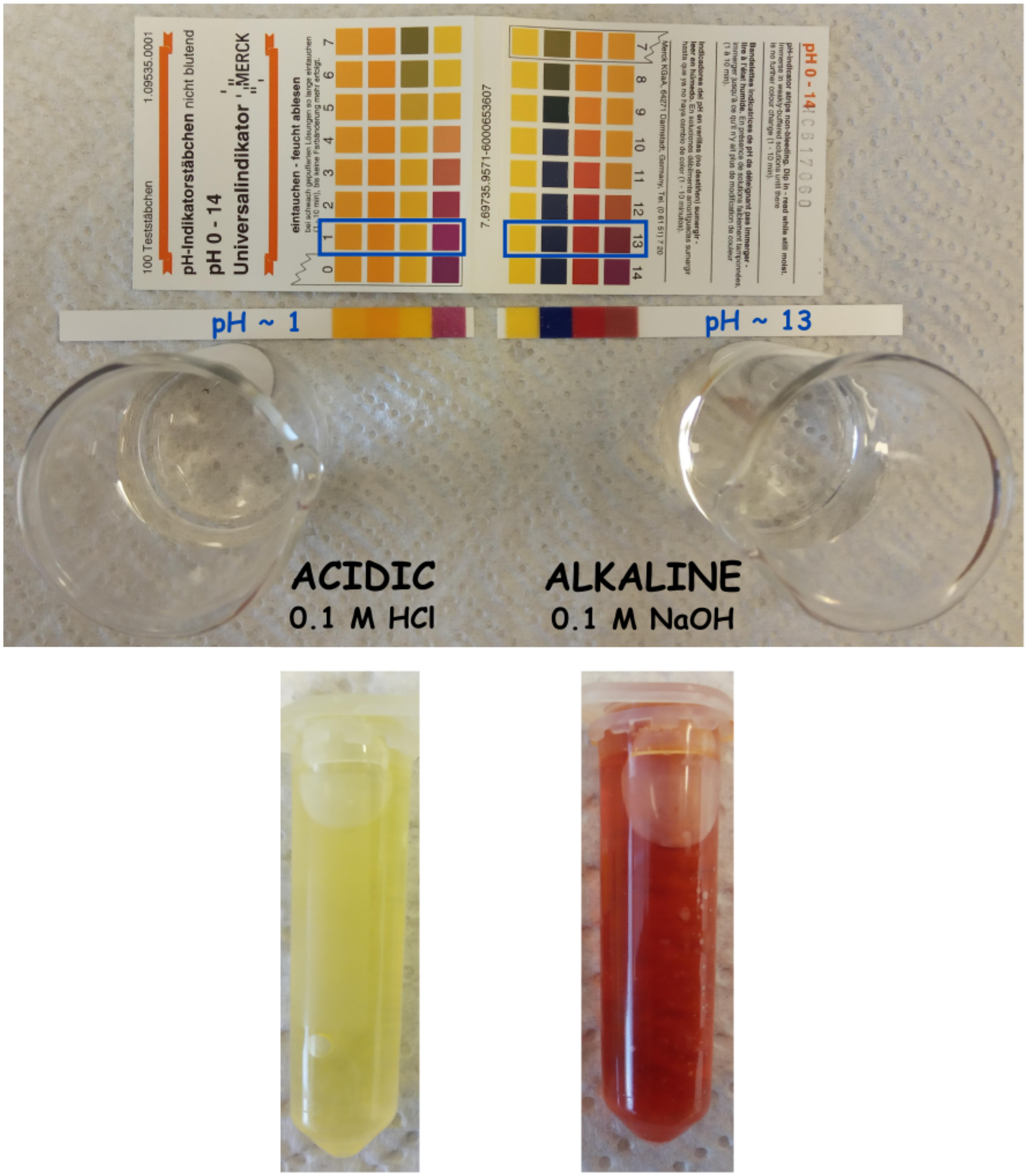

Figure S5: *Top:* Determination of the pH values of the HCl and NaOH solutions using pH stripes. *Bottom:* Colors of the turmeric spice in acidic and alkaline media.

5 Electrochromic Device Assembly

1. *Electric wires:* Two stripes of 2 cm $\times$ 15 cm dimension were cut from the kitchen aluminum foil. They were rolled into a cylinder. An insulation tape was placed over the surface to insulate the whole cylinder except for the furthermost 0.5 cm on each end. (Figure S6/2).

2. *Electrodes:* Two identical clean non-sharpened stainless steel kitchen knives were first covered on one side of their non-cutting surface by insulation tape. The whole metallic surface was covered. Excess tape was cut away with scissors. (Figure S6/3) The knives were put together so that the two insulated surfaces were touching and the knife holders were placed on the opposite sides. Insulation tape was used to secure the assembly (tip of one knife and the holder of the other knife were glued together on each side of the assembly). (Figure S6/4)

3. *Mounting the EC carrier:* A stripe of the dry impregnated paper towel was slowly rolled across the exposed metal surface of the assembly. It was tightly pressed onto the surface of the knives (Figure S6/7) and held in place with an office paper clip.

4. *Soaking with an electrolyte solution:* Using a dropper, the EC carrier was soaked with the aqueous solution of baking soda. When wet, the paper clip was removed. (Figure S6/8)

5. *Encapsulation:* A transparent office adhesive tape was slowly rolled over the wet EC carrier, starting on the one side of the device and proceeding to the other knife holder. (Figure S6/9)

6 *Connecting the device to a battery:* One end of the first wire was attached to a 4.5 V alkaline battery and one end of the second wire as attached to the other pole of the battery. Insulation tape was used to secure the connection. The free ends of the two wires were connected to metal surfaces of the respective knife holders. (Figure S6/11) The wires were kept in such positions that the unprotected metal surfaces never touched (preventing short circuits).

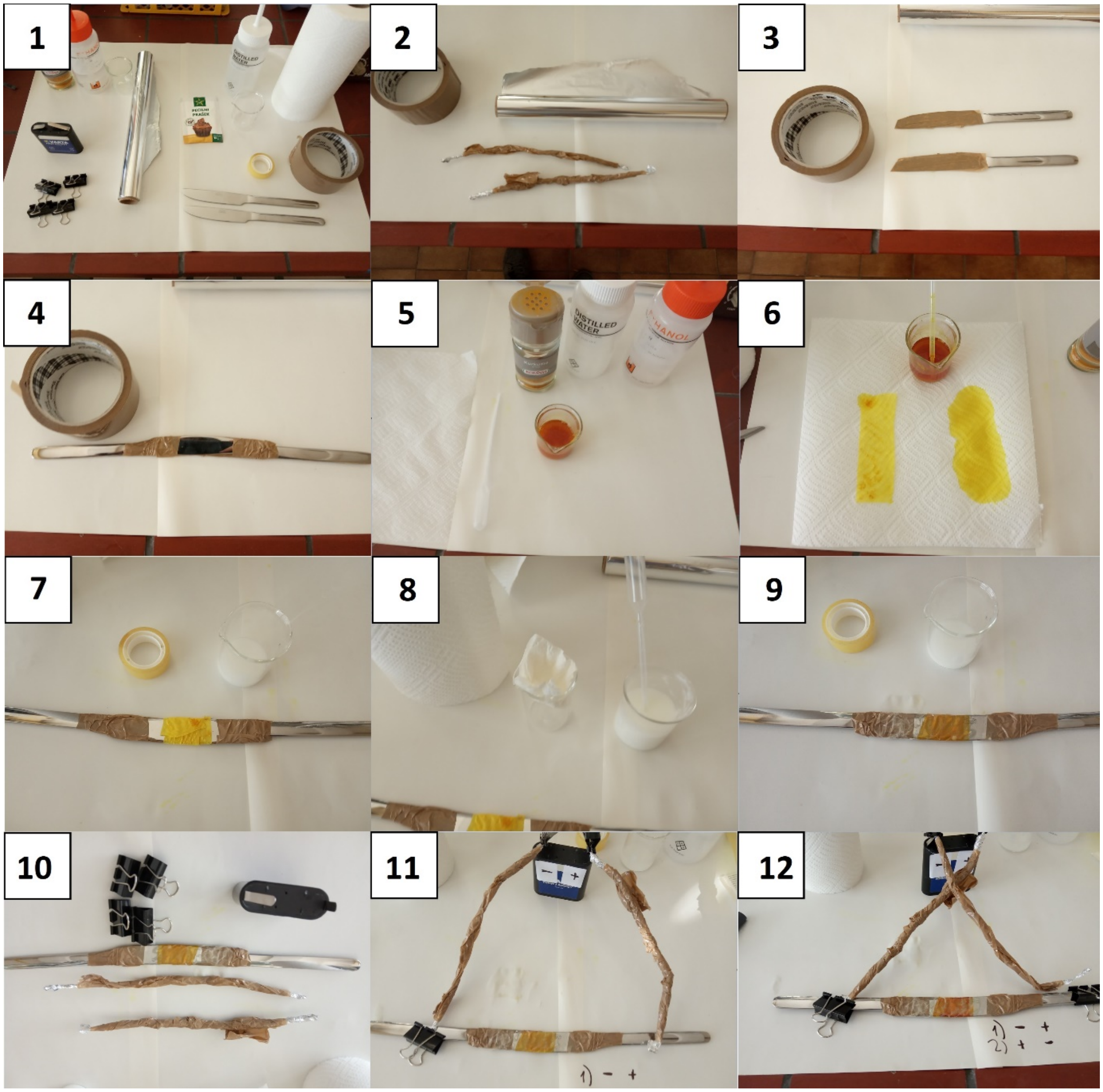

Figure S6: Steps of electrochromic device assembly (1-11) and testing (11 & 12).

6 Device Testing

A new 4.5 V alkaline battery was used. When connecting the electrochromic device to the battery, the time for a warm-up cycle was first measured with a stopwatch. Then the office clips on the two electrodes were removed and wires were reconnected to the opposite sides of the device (Figure S6/11 and 12). The time of the first working cycle on the visible electrode was measured. Then the polarity was reversed again and the time for the 2nd working cycle was recorded. Polarity was reversed once more, and time for the 3rd working cycle was measured. The times of the warm-up cycle and of the first three working cycles with indicated polarity alongside with the color of the visible surface of the electrochromic device are presented in a Table S6.

Table S6: Times for the warm-up cycle and for the first three working cycles on a viewable electrode with indicated polarity. The color of the viewable surface of the electrochromic device is indicated.

cycle	electrode polarity	time [s]	observed color
warm-up	$+$	44	yellow
1st	$-$	109	orange-red
2nd	$+$	109	yellow
3rd	$-$	112	orange

The working area of the electrodes was 19 mm $\times$ 19 mm = 361 mm^2.

7 Remarks

During the device operation, gas bubble formation was observed on the sides of the electrodes (where the tape was attached to the metal). Moreover, the coloration was not entirely uniform on the whole visible surface and several small color spots formed during cycling. These were most likely due to trapped bubbles, which prevented a uniform change of the solution's pH value.

8 Conclusions

The reported experiment demonstrates that ECDs can be constructed without optically transparent electrodes from very basic household materials. It is possible to control the pH at the visible side of the electrode, with the goal of inducing the pH change and thus coloration change of the device. We can also reversibly change coloration to the desired color and keep it even after the device has been disconnected.

Supporting Information (IV)

Student Questionnaire for the
Electrochromic Device Demonstrator
from Household Materials

Name: ________________________ **Date:** ________________________

1. What is electrochromism?

(A) Electrochemical deposition of chromium onto a metal surface.

(B) Change in color or opacity of a material due to an external voltage or current, where the material retains the existing color even after being disconnected from the power source.

(C) Preparation of polychromatic plastics.

(D) Change in coloration due to change of orientation of liquid crystals under the influence of an electric field.

2. What causes the color changes of an aqueous solution of a given pH indicator?

(A) Variation in the concentration of the H^+ (or H_3O^+) ions in the solution.

(B) Variation in the oxidation state of the atom, composing the indicator molecule.

(C) Variation in the concentration of metal ions.

3. During the electrolysis of neat water the pH value near the anode and cathode changes. Which statements are true for the process occurring at the anode?

(A) The solution is becoming negatively charged.

(B) The solution turns alkaline.

(C) $2H_2O \longrightarrow O_2 + 4H^+ + 4e^-$

(D) $2H_2O \longrightarrow O_2 + 2H_2$

4. Sketch the basic architecture of the inverted-sandwich electrochromic device (ECD) and name its components. Indicate the current flow. What are the respective charge carriers in the metal and in the electrochromic solution?

5. What was the purpose of the baking soda solution used in the constructed ECD?

(A) To increase the electrical conductivity of water.

(B) To produce CO_2 gas during electrolysis.

(C) To change the color of turmeric spice.

6. What is a response (cycling) time of an ECD?

(A) Time needed to connect an ECD to a battery.

(B) Time needed for an ECD to change between the two differently colored states.

(C) Life-time of an ECD.

7. What is the difference between electrolytic cell and ECD?

(A) The electrolytic cell is used for the production of materials (e.g., chlorine gas, aluminum), while ECD can be used as a method to change the color of the surface (screen) or as a smart window.

(B) All electrolytic cells are similar to ECDs in that they can be used for simple experiments only.

7. Describe your observations and conclusions from the experiment you conducted. What components could you replace with other materials (different dye, different electrolyte etc.)?

Answers

1B; 2A; 3C; 5A; 6B; 7A

4

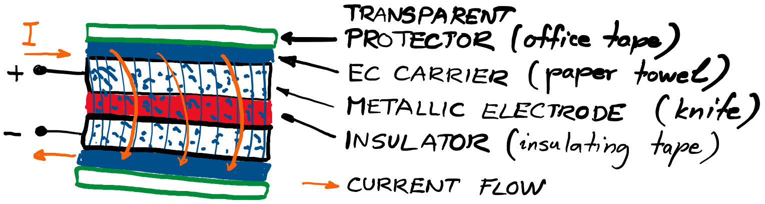

Figure S7: Basic architecture of the inverted-sandwich ECD and its components. Orrange arrows indicate current flow.

Metal charge carriers - electrons
Solution charge carriers - ions

When connected to an external power supply, anode is positively charged electrode (the solution becomes acidic in the vicinity) while cathode is negatively charged electrode (the solution becomes basic in the vicinity). Near anode ther solutions turns orange to yellow, while near cathode it turns orange to red.

8

- Any observations as to why the device works or does not work, and guesses as to why the device works the way it does (no color change - could be due to bubbles or short circuit etc.).
- Use of Indigo carmine found in pen ink, use of liquid or solid soap as electrolyte etc.

Score statistics

One point was awarded for a correct answer to each of the eight questions (the total number of points was 8). Figure S8 shows the number of students who scored a certain number of points. The total number of students who took the tests was 14.

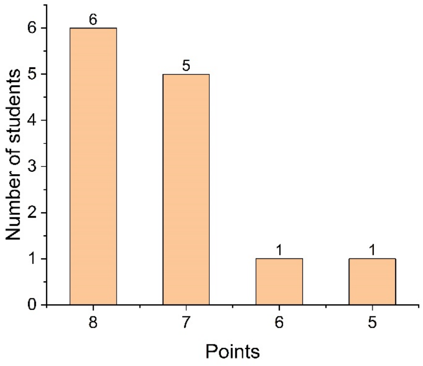

Figure S8: Test performance.
